# The Potential of Optimized Liposomes in Enhancement of Cytotoxicity and Apoptosis of Encapsulated Egyptian Propolis on Hep-2 Cell Line

**DOI:** 10.3390/pharmaceutics13122184

**Published:** 2021-12-17

**Authors:** Enas Alaa El-din Abd El-aziz, Sherif Farouk Elgayar, Fatma M. Mady, Mohammed A. S. Abourehab, Omiya Ali Hasan, Lamis M. Reda, Eman Alaaeldin

**Affiliations:** 1 Department of Oral and Maxillofacial Pathology, Faculty of Dentistry, Minia University, Minia 61519, Egypt; enasalaaeldin@yahoo.com (E.A.E.-d.A.E.-a.); selgayar@gmail.com (S.F.E.); 2 Department of Pharmaceutics, Faculty of Pharmacy, Minia University, Minia 61519, Egypt; fatmamady@hotmail.com (F.M.M.); mohawahab2002@yahoo.com (M.A.S.A.); 3 Department of Pharmaceutics, Faculty of Pharmacy, Umm Al-Qurra University, Makkah 21955, Saudi Arabia; 4 Department of Pharmaceutics and Industrial Pharmacy, Faculty of Pharmacy, Deraya University, Minia 61768, Egypt; omaya.ali@deraya.edu.eg; 5 Department of Pharmaceutics and Clinical Pharmacy, Faculty of Pharmacy, Sohag University, Sohag 82524, Egypt; 6 Department of Clinical Pharmacy, Faculty of Pharmacy, Deraya University, Minia 61768, Egypt; lamismohamed06@gmail.com

**Keywords:** propolis, liposomes, Hep-2, apoptosis, cytotoxicity

## Abstract

Purpose: Development of pharmaceutical dosage forms of natural products has gained great interest recently. Propolis is a natural product with various active compounds and multiple pharmacological activities. Its resinous nature and low bioavailability were obstacles in the optimum use of this magnificent natural product. Aim: This study evaluates the effect of using liposomes as a drug delivery system on the enhancement of the cytotoxic effect of propolis on squamous cell carcinoma cell lines (Hep-2) of head and neck. Methods: An optimized liposomal formulation of propolis was prepared using the conventional thin film hydration method 1, 2. The prepared (Hep-2) cell line was treated with different concentrations of propolis and optimized propolis liposomes for 24 h. The effect of both propolis and propolis liposomes on cell line was investigated using MTT assay, cytological examination, and nuclear morphometric analysis. The effect of the drugs on the cell apoptosis was evaluated using Annexin V. Results: The findings revealed that both propolis and propolis liposomes have a cytotoxic effect on Hep-2 cell line through induction of apoptosis. The effect was dose dependent. However, a statistically significant enhancement in propolis-mediated apoptosis on Hep-2 cells was elucidated due to encapsulation within the prepared liposomes. Conclusion: Liposome is a powerful tool for enhancing the cytotoxicity of propolis against Hep-2 cell line.

## 1. Introduction

Head and neck cancer (HNC) is a term used to define malignant neoplasms that originate from the oral cavity, nasal cavity, larynx, pharynx, and paranasal sinuses [1]. HNC can affect and impair the quality of life of patients through the interruption of important daily functions including: breathing, speaking, and swallowing. Moreover, they cause physical and emotional downsides for the patient [2]. According to the World Health Organization, the oral cavity is the most frequently affected part, with about 389,000 cases every year [3].

Tobacco and alcohol are responsible for about 75% of HNC cases in USA and Europe [4]. An alcoholic beverage (three times daily) doubles the risk of developing the disease. In addition, passive smoking gives the same risk of developing the disease as first-hand smoking [1]. Alcohol and tobacco simultaneously increase the risk of the development of HNC 35-fold [5]. Human papillomavirus (HPV) also has a significant effect on the development of HNCs [6], particularly HPV 16 and 18 [7].

Despite the various techniques that have been developed for treatment of HNC, alternative strategies that can maintain long survival rates with minimal adverse effects are still needed [8]. Recently, great attention has been paid to natural substances or products as a promising source for new anticancer strategies. Over 70% of anticancer compounds are represented as natural products or substances derived from nature such as propolis. Cytotoxic natural products conjugated to polymeric carriers or monoclonal antibodies produce more efficient targeted remedies [9].

Propolis has been recently widely investigated for its antitumor effect using in vitro and in vivo experimental models. It contains many compounds such as flavonoid aglycones polyphenols, phenolic aldehydes, and ketones. Those compounds are well known to have anticarcinogenic activity [10]. Overall, propolis is a honeybee product with immunomodulatory, antioxidant, anti-inflammatory, bactericidal, antiviral, and antiparasitic activities [11]. The chemical composition and proportions of different constituents of propolis extract depend on the geographic diversity of bee species and plant sources [12,13,14]. Caffeic acid, phenethyl ester (CAPE), and many other flavonoids and phenolic compounds are the major constituents of the Egyptian propolis [13].

The antitumor activity of propolis is mediated by immunomodulatory action [12]. This is achieved through macrophage activation, which leads to the augmentation of non-specific antitumor immunity with the production of soluble factors that interfere directly with the tumor tissue or in the mode of action of other immune cells [13]. It was found that flavonoids are the compounds responsible for many of the biological activities of propolis [14]. Despite the promising activities of such flavonoids, their instability, resinous nature, and low bioavailability are facts that limit the use of propolis [15].

The valuable targeted drug delivery system of liposomes has been introduced for being biodegradable, biocompatible, non-toxic, and its capability of encapsulating both lipophilic and hydrophilic drugs [16,17]. Those unique characteristics of liposomes guarantee the encapsulation of the hydrophilic and lipophilic components of natural extracts. Moreover, they ensure targeting and enhanced cell utilization due to the small size of drug-loaded vesicles [18]. Propolis flavonoids can be encapsulated with liposomes, thus increasing their stability and improving their actions.

The present study aimed to prepare propolis-encapsulated liposomes and investigate the possible enhancement effect of encapsulation within liposomes on the anticancer effect of propolis towards HEP-2 cell line.

## 2. Materials and Methods

### 2.1. Materials

Drug: Propolis ethanolic extract was bought from the department of Cell Culture, VACSERA-EGYPT (net content of 72.95%). It was prepared from the Egyptian propolis by extraction with absolute ethanol in a closed glass container for 4 days at 37 °C, with shaking. Then, filtration of the ethanolic extract was performed and concentrated by evaporation in a rotary evaporator, under reduced pressure at 60 °C. Ethanolic extract of propolis (EEP) was dissolved in DMSO (50 mg·mL^−1^), and the final concentration of DMSO in the culture medium was adjusted at 0.1% (*v*/*v*). Chloroform and cholesterol (>99%) were obtained from Sigma-Aldrich (St. Louis, MO, USA). Phosopholipon 90 H^®^ (PL 90 H), a purified and granulated soy lecithin with hydrogenated phosphatidylcholine content of 90%, was donated by Lipoid GmbH Germany. Deionized water was obtained from the microbiological laboratory for water and food analysis (Minia, Egypt). All other materials were of analytical grade.

### 2.2. Methods

#### 2.2.1. Preparation of Propolis-Loaded Liposomes

The conventional thin-film hydration method [19,20] was used to prepare the propolis-loaded liposomes (P-Lip). In brief, propolis and the specified amount of PL 90 H^®^ were dissolved in the least volume of chloroform with different molar ratios (Table 1). The organic solvent was evaporated at 37 °C using a rotary evaporator under reduced pressure (Stuart, RE300 Germany) at 110 rpm until the formation of a dried thin film of the lipid propolis mixture on the wall of the rotating flask. The formed film was kept for 24 h in a desiccator to eliminate any organic solvent. A calculated amount of (9% *w*/*v*) sucrose solution was used to hydrate the formed film. The rotating flask was kept rotating in a water bath under normal pressure at 37 °C for 2 h until complete hydration of the film. The formulated liposomes were remained overnight at 4 °C to permit the strengthening of the lipid bilayer [21]. Then, the liposomal formulations were available for additional evaluation and in vitro characterization processes [21].

#### 2.2.2. Determination of Entrapment Efficiency (EE%)

The percentage of total flavonoids encapsulated within the prepared liposomes was detected indirectly by subtracting the unencapsulated amount from the initial amount of the drug [22]. First, 1 mL of the prepared P-Lip was centrifuged for one hour at 4 °C for removal of the unentrapped drug from the formed liposomes using a cooling centrifuge (HermLe^®^ Z326 K) (Wehingen, Germany). Washing by resuspension of liposomes in deionized water was performed in order to confirm a full removal of the unentrapped drug. The prepared liposomes were then centrifuged again. A spectrophotometric assay was performed on the separated supernatant each time [23]. Quantitative analysis is based on the colorimetric analysis of total flavonoid content reported by Woisky and Salatino [24]. Briefly, 50 µL of 10% alcoholic solution of aluminum chloride was mixed with 50 µL of the supernatant and made to a volume of 1 mL using absolute alcohol. Absorbance was measured at λ = 410 nm (Spectronic Genesys^®^, with Winspec Software, Spectronic, Melville, NY, USA). Entrapment efficiency (%) was determined based on the following equation [22]:

(1) $$\mathrm{EE}\%=\frac{\mathrm{Initial}\;\mathrm{amount}\;\mathrm{of}\;\mathrm{flavonoids}\;\mathrm{added}\;-\;\mathrm{unentrapped}\;\mathrm{flavonoids}}{\mathrm{Initial}\;\mathrm{amount}\;\mathrm{of}\;\mathrm{flavonoids}}\times 100$$

#### 2.2.3. Evaluation of Particle Size and Zeta Potential

The prepared liposomes were diluted with purified deionized water, particle size and size distribution were evaluated at 25 °C using Mastersizer (3000E, Malvern Instruments, Malvern, UK), and average values after triplicate repetition of each preparation were used [25]. The zeta potential of Millipore water-diluted samples was determined. The average zeta potentials were calculated [26]. The morphological features of the formed liposomes were examined using transmission electron microscope (TEM) (JM 1000 EX, Peabody, MA, USA). Briefly, a sample was mounted on hydrophobic grids, dried, and then stained with uranyl acetate (50 µL, 2.5% *w*/*v*).

To gain more insight into the stability of formulated liposomes, selected formulation (F4) was evaluated for any change in size and/or EE% after 3 and 6 months of storage at 25 °C.

#### 2.2.4. In Vitro Release Study

In vitro release of propolis from the formulated liposomal systems was examined using the dialysis membrane method. In brief, a sample of P-Lip formulations with equivalent flavonoid concentrations was placed in pre-soaked dialysis bags. These bags were kept floating on the release media, herein, phosphate buffer (pH 7.4), containing 0.5% tween 80 to preserve sink condition [27]. The system was stirred at 50 ± 10 rpm using a thermostatic shaker at 37 ± 0.5 °C. Then, 2 mL samples were withdrawn at predetermined time intervals over a period of 6 h and were replaced with an equal volume of fresh medium. Flavonoidal concentration was detected using the aforementioned Woisky and Salatino technique [24]. The evaluation was repeated thrice, and the average values were calculated and recorded [28].

The release kinetics of the studied liposomal formulations were investigated by fitting the permeation data to the following models:

Zero-order: R = K_0_.

First-order: R = 1 − e^−k^_1_. t

Higuchi diffusion model: Q = K_H_. t^1/2^,

where R or Q is the fraction of drug permeated at time t, K, or KH is the rate constant corresponding to each model.

#### 2.2.5. Cytotoxicity Assay

Cytotoxicity against laryngeal squamous cell carcinoma cell line Hep-2 cells VACSERA-EGYPT) was performed by 3-[4,5-dimethylthiazol-2-yl]-2,5 diphenyltetrazolium (MTT) assay. The cells were grown in MEM-H (InvitrogWaltham, MA, USA) as a culture medium containing 10% fetal bovine serum (GIBO COBRAL^®^ Limited, Edinburgh, Scotland), 4 mM^−l^ glutamine, 100 U·mL^−1^ penicillin, and 100 μg·mL^−1^ streptomycin, then incubated at 37 °C in an atmosphere containing 5% CO_2_. Hep-2 cells were seeded for 24–48 h before the experiments onto a 96-well plate. The cells were treated with serial concentrations of propolis and P-lip (F4) for 24 h. The medium was removed, and then MTT solution (20 μL, 5 mg·mL^−1^) (Sigma Chemical Company, St. Louis, MO, USA) was added to each well for 4 h. DMSO was used to dissolve the formed crystals. Hep-2 cells and medium alone represented the control. The spectrophotometric absorbance was measured using Dynatech MR5000 spectrophotometer (Dynatech Laboratories, Inc., Chantilly, VA, USA) at 550 nm. The percent cytotoxicity was calculated by the formula:

Percent viability (viable cell%) = ([absorbance of experimental wells/absorbance of control wells]) × 100%

Results were determined by trying three independent experiments. Data were analyzed with Master Plex Reader Fit program to estimate the IC_50_, the half maximal inhibitory concentration, of both propolis and P-lip.

#### 2.2.6. Cytological Evaluation and Nuclear Morphometric Analysis

Hep-2 cells were managed with half IC_50_, IC_50_, and double IC_50_ of propolis and P-lip (F4) for 24 h. Treated cells were dispended and fixed on clean glass slides.

For cytological examination. Ten fields of each slide were photomicrographed at the power of 1000X oil. The images were analyzed on a computer system using image analysis software (Image J, 1.27z, NIH, Bethesda, MD, USA). The surface area and nuclear circularity were measured. Nuclear area factor (NAF) was determined using the formula [29]:

NAF = Circularity × Object area

#### 2.2.7. Evaluation of Apoptotic Cell Death Using Annexin V-FITC Staining

Hep-2 cells were seeded in 24-well plates for 24–48 h and then exposed to propolis or P-lip for other 24 h. Cells were rinsed with PBS for two times and suspended in 1 mL of binding buffer. A certain volume of cell suspension (250 μL) was incubated with 2.5 μL of annexin V-FITC and 5 μL propidium iodide (PI) at ambient temperature and darkness for 10 min. Apoptotest-FITC Kit (Dako, Glostrup, Denmark) was used for Annexin V assay. The V-positive population of annexin was estimated using flow cytometry (BD FACScan, Becton Dickinson Immnunocytometry Systems, San Jose, CA, USA).

#### 2.2.8. Statistical Analysis

All results are recorded as mean ± SD of three separate experiments. Student’s *t*-test was used to compare between means of two groups. One-way ANOVA test with Bonferroni post hoc test was used to compare means of more than two groups. *p*-values ≤ 0.05 were considered significant.

## 3. Results

### 3.1. Preparation of Propolis Liposomes

**Figure 1:**
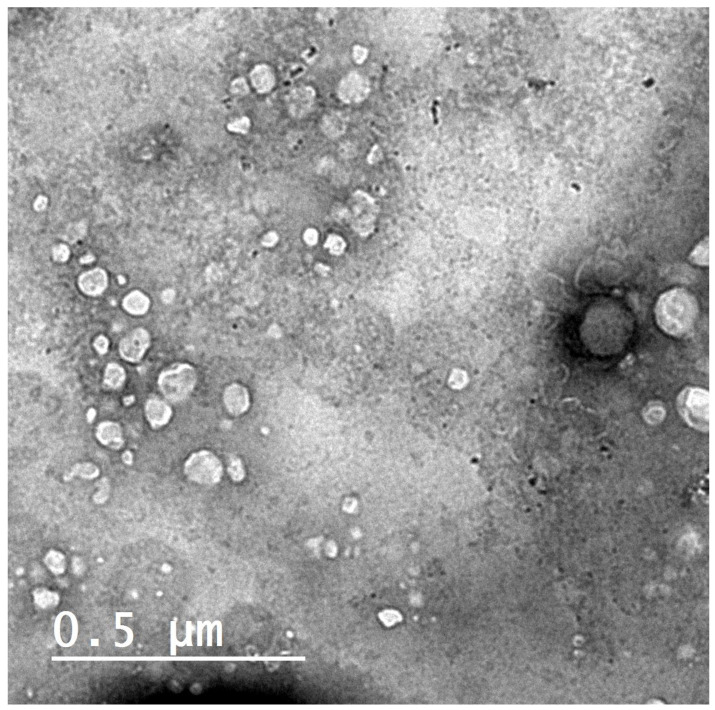
Transmission electron micrograph of the prepared liposomes P-lip (F4) when stained with uranyl acetate (2.5% *w*/*v*) with scale 0.5 µm.

Liposomes (F4) visualized by transmission electron microscopy appeared to be predominantly spherical Figure 1. The figure shows the homogenous size distribution, which supports the low PDI of F4, as shown in Table 1.

### 3.2. Effect of Formulation Parameters on Entrapment Efficiency (EE%) and Particle Size

The effect of various formulating parameters on EE% of propolis liposomes is shown in Table 1. The results show that the molar concentration of lipid (MCL) significantly affects the EE% of propolis. The EE% of propolis significantly increased from 63.2 ± 1.5% up to 85.3 ± 3.4% when the MCL increased from 40 mM mL^−1^ (F2) to 80 mM mL^−1^ (F3) (*p* < 0.001). Cholesterol molar concentration showed a positive effect on propolis EE%. Table 1 shows that entrapment efficiency was significantly increased from 66.3 ± 1.6% to 72.9 ± 2.8% when cholesterol molar concentration increased from 20% mol/mol (F4) to 40% mol/mol (F1) (*p* < 0.001).

The particle size values of the freshly prepared liposomes were in the range of 126.5 ± 3.4 nm and 723 ± 20.5 nm (Table 1). The smallest particle size was recorded with F4, while the largest value was found with F3. The wide variation in particle size values may be due to the distinct cholesterol content and drug loading [30]. Different cholesterol% and consequent different DL amount may be the reason for the different particle size of the formulated vesicles. Increasing cholesterol% tends to increase drug loading, which results in increase vesicle size and vice versa. Zeta potential is the electrostatic charge of the vesicle surface, which acts as a repulsive force controlling the stability of liposomes and other dispersions and opposing the proximity of liposomal vesicles and aggregation. Zeta potential values of the prepared liposomes (F1–F5) presented in Table 1 were in the range of −13.1 ± 1.4 and −20.2 ± 3.2 mV. These findings confirmed that all formulations are almost stable [31].

With up to 6 months of storage of the selected F4 liposomal formulation, no significant change in the EE% was detected (Table 2). There was substantial increase in the particle size after 6 months, which may be attributed to the tendency of some vesicles to fuse together; however, the change in size is still accepted and reveals the stability of the formulated liposomes.

### 3.3. Effect of Formulation Parameters on the Release

**Figure 2:**
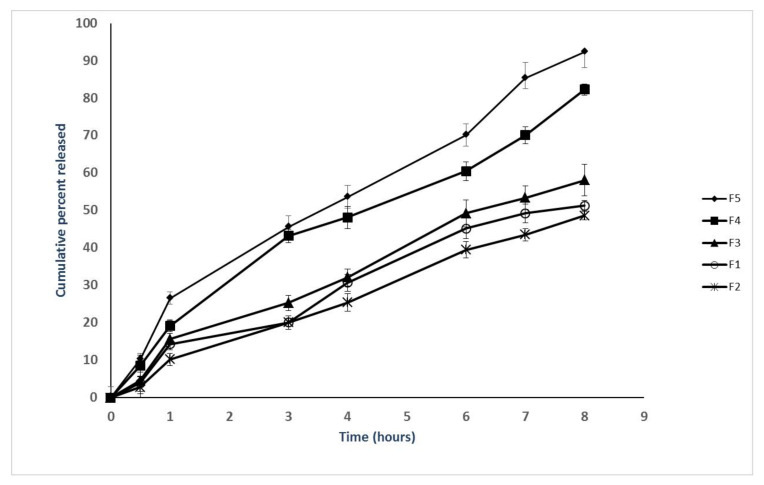
In vitro release of flavonoids from the prepared propolis-encapsulated liposomes (F1–F5) in phosphate-buffered solution containing 0.5% tween 80 to maintain sink condition (PBS, pH 7.4, 37 °C). The results are represented as the means ± SD (*n* = 3).

In vitro release of flavonoids from the prepared propolis-encapsulated liposomes (F1–F5) was represented in Figure 2. The results show that cumulative amounts of flavonoids released within 8 h could be arranged in descending order as follows: F5 > F4 > F3 > F1 > F2. The low content of cholesterol (20%) in the prepared propolis encapsulated liposomes (F5 and F4) is responsible for the increased release of the entrapped flavonoids due to the reduced rigidity and the increased fluidity of the formed bilayer. Moreover, higher drug loading of the prepared propolis-encapsulated liposomes (F5, F4) is another factor leading to increased release of flavonoids. However, higher content of cholesterol (40%) in the prepared propolis-encapsulated liposomes (F1, F2, and F3) makes the prepared liposomes more rigid with delaying the release of encapsulated flavonoids [31]. The formulated liposomes exhibited higuchi release kinetics, which is suitable for such dispersion system (Table 3).

### 3.4. MTT Assay

**Figure 3:**
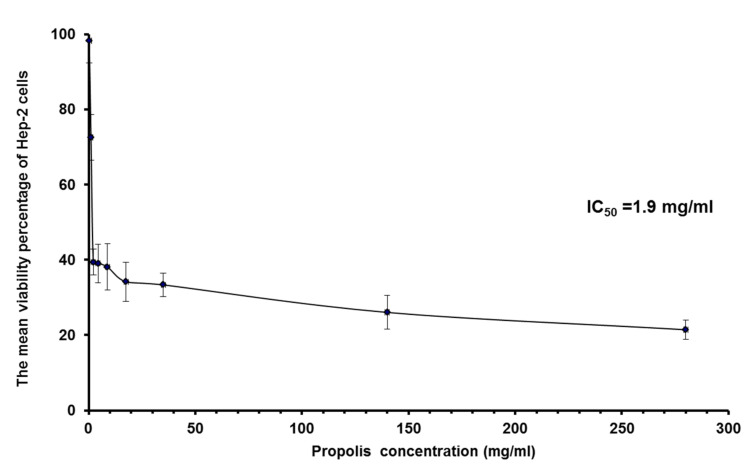
The mean viability percentage of HEP-2 cells treated with different concentrations of propolis extract.

**Figure 4:**
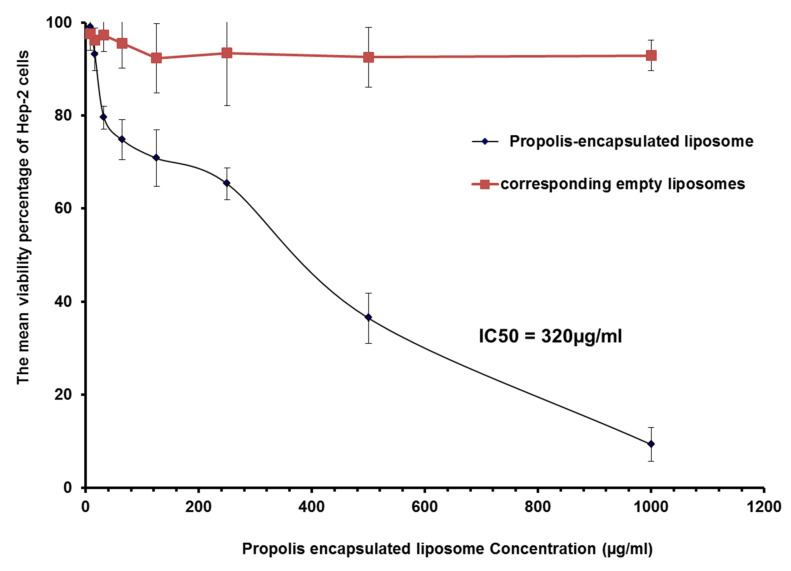
The mean viability percentage of HEP-2 cells treated with different concentrations of liposomal propolis and corresponding empty liposomes for 24 h.

The viability percentage of Hep-2 cells treated with increasing concentrations of propolis or propolis-encapsulated liposome (F4) for 24 h was listed in Table 4 and Table 5 (Figure 3 and Figure 4). F4 was selected for MTT assay, because it exhibited minimal particle size with enhanced EE% and release%.

Both propolis and liposomal propolis inhibited the growth of Hep-2 cells. Their effects were more prominent as the concentration increased. IC_50_ of propolis and propolis-encapsulated liposomes were calculated as 1.9 mg/mL and 0.32 mg/mL, respectively. These results revealed that liposomal propolis had a more cytotoxic effect after incubation for 24 h. Empty liposomes did not show any anti-proliferative effect, as shown in Figure 4.

### 3.5. Cytological Evaluation and Nuclear Morphometric Analysis

**Figure 5:**
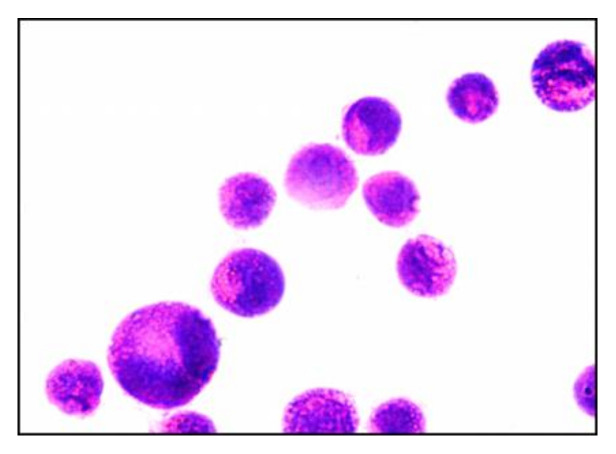
Micrograph of untreated Hep-2 cells (control cells) at the power of 1000X oil.

Microscopic examination of control cells revealed the presence of criteria of malignancy as relatively regular, hyperchromatic, and condensed nuclei. The cellular outline was also relatively regular with minimum folding in the cellular or nuclear membrane. Only a few cells showed morphological criteria of apoptosis as nuclear fragmentation, as shown in Figure 5.

**Figure 6:**
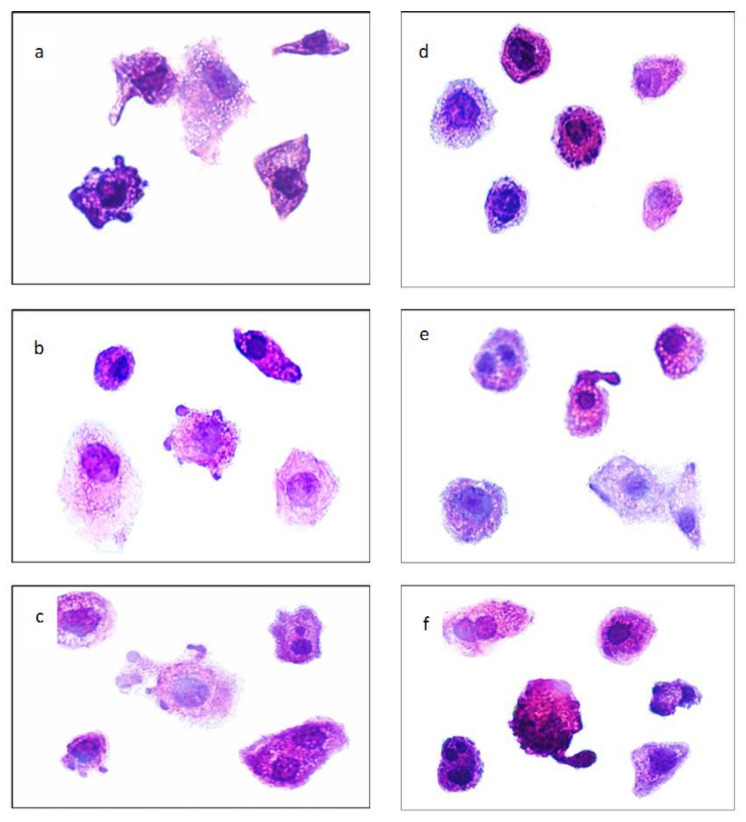
Micrograph showing criteria of apoptosis in Hep-2 cells treated with increasing concentrations of propolis (half IC_50_ of propolis (**a**), IC_50_ of propolis (**b**), double IC_50_ of propolis (**c**)) or propolis encapsulated-liposome (half IC_50_ of propolis-encapsulated liposome (**d**), IC_50_ of propolis-encapsulated liposome (**e**), and double IC_50_ of propolis encapsulated-liposome (**f**)) for 24 h at the power of 1000X oil.

On the other hand, treated cells showed nuclear morphological alterations that matched with morphological criteria of apoptosis in its different stages. These criteria included peripheral condensation of chromatin against nuclear membrane (margination), irregularities in the nuclear and cellular membrane, nuclear shrinkage, nuclear segregation, nuclear fragmentation, and apoptotic bodies formation. In addition to these apoptotic criteria, fewer cells showed nuclear changes that matched with morphological changes of necrosis; characteristically coarse staining and clumping of the heterochromatin admixed with euchromatin with relative preservation of nuclear morphology, nuclear and cellular swelling, and rupture of the cell membrane, as shown in Figure 6a–f.

Some of these morphological criteria of alterations, including apoptosis and necrosis, were more obvious and more common in cells treated with P-Lip.

Regarding morphometric analysis, data recorded revealed that HEp-2 cells treated with different concentrations of propolis showed a decrease in the mean value of NAF with increasing propolis concentration. A much more significant decrease in the mean value of NAF was observed when Hep-2 cells were treated with liposomal propolis. These findings matched with what was found in the cytotoxicity study.

### 3.6. Apoptotic Cell Death Assay

**Figure 7:**
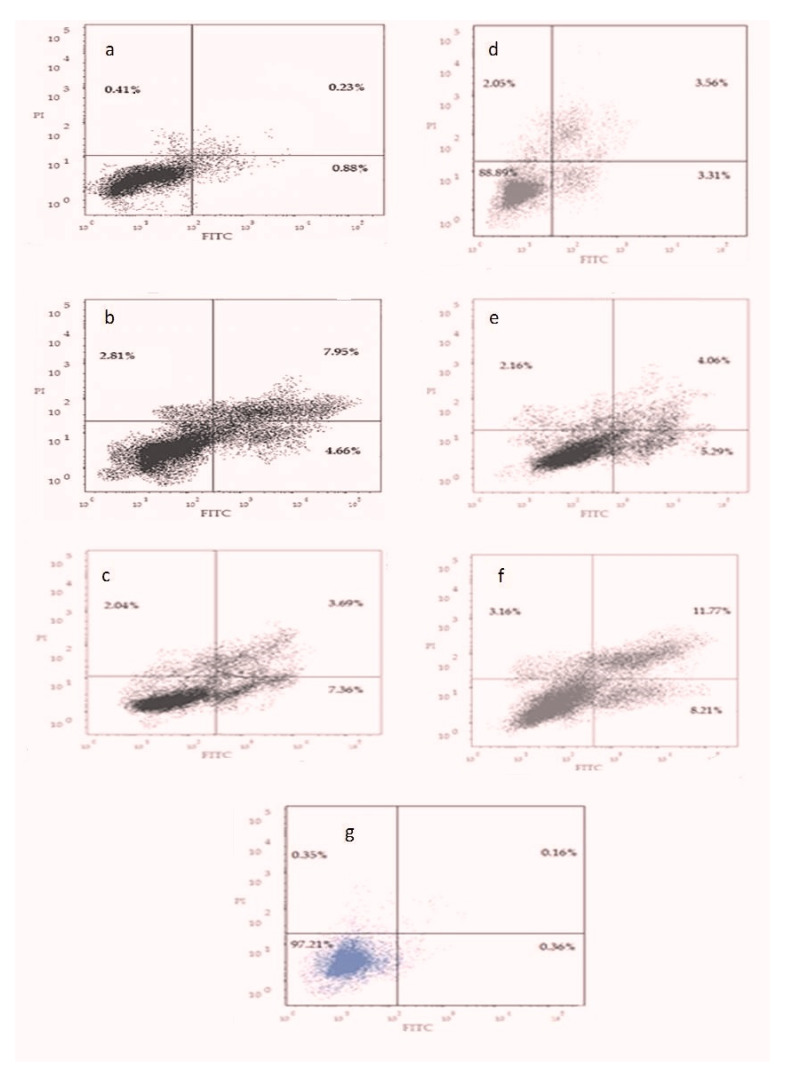
Annexin V-FITC and propidium iodide. A contour plot for cells treated with different concentrations of Propolis (half IC_50_ of propolis (**a**), IC_50_ of propolis (**b**), double IC_50_ of propolis (**c**)) or propolis-encapsulated liposome (half IC_50_ of propolis-encapsulated liposome (**d**), IC_50_ of propolis-encapsulated liposome (**e**), and double IC_50_ of propolis-encapsulated liposome (**f**)), and control cells (**g**).

The majority of control cells were viable and non-apoptotic. Increasing the concentration of propolis resulted in a decrease in the percentage of viable cells and an increase in cells undergoing early apoptosis. The maximum effect was evident with IC_50_ concentration of propolis. A slight increase in the necrotic cells was observed with increasing propolis concentrations, as shown in Figure 7a–c, in comparison to control cells, as shown in Figure 5.

Treating the cells with P-Lip resulted in increasing the percentage of apoptotic cells with maximum effect recorded with double IC_50_ concentration of P-Lip, as shown in Figure 7d–f.

### 3.7. Statistical Analysis

#### Effect of Different Concentrations of Unencapsulated Propolis on NAF

Mean values of NAF of Hep-2 cells treated with different concentrations of propolis and the control cells were significantly different, as determined by ANOVA test, Table 6.

Mean values of NAF of Hep-2 cells treated with different concentrations of propolis were significantly different from those of the control cells as revealed by post hoc multiple comparison test (Turkey HSD). However, a statistically insignificant difference was observed when comparing the mean value of NAF of Hep-2 cells treated with various concentrations of propolis extract, as presented in Table 7 and Table 8.

### 3.8. Effect of Different Concentrations of Propolis-Loaded Liposomes on NAF

Regarding the mean of NAF of Hep-2, the ANOVA results showed a significantly difference values between different concentrations of P-Lip-treated cells and the control cells, as presented in Table 6.

Post hoc multiple comparison test (Turkey HSD) revealed a statistically significant difference among mean values of NAF of different concentrations of P-Lip-Hep-2-treated cells and the control cells. Moreover, NAF values of Hep-2 cells treated with half IC_50_ concentration, and those treated with double IC_50_ of P-Lip were significantly different, as presented in Table 9 and Table 10.

### 3.9. Comparison between the Effect of Unencapsulated Propolis and Encapsulated Propolis on NAF

Independent sample *t*-test showed significant difference when comparing the mean value of NAF of propolis-treated Hep-2 cells and that of cells treated with P-Lip, as presented in Table 11.

## 4. Discussion

An ideal antitumor drug is an agent that not only can destroy cancer cells but also should be safe and produce a maximum effect at a lower dose with minimal side effects.

Propolis is a fertile source for the discovery of new pharmaceuticals due to its biological and pharmacological properties, which are correlated to its flavonoid content. However, although flavonoids and other components of propolis are of various useful pharmacological activities, the optimum application of this natural grant is hindered by the instability [14], limited water solubility, and resinous nature of the propolis extract [32]. This has motivated researchers to find a formulation of propolis able to maintain biological properties and structural integrity and improve its water solubility and bioavailability, which can be achieved by the use of liposomes [16,33,34,35].

An attempt of the optimization of liposomal formula of propolis extract in terms of enhanced EE% and reduced particle size was carried out, maintaining variable formulation parameters including DL, CH%, and MCL. The results show the increased EE% with increasing MCL, which may be attributed to the increased size of the lipid bilayer, which allows the entrapment of more lipophilic drugs [33,35,36]. The increased CH% also increased EE%, which may be attributed to the improved stability of the formulated liposomes. The rigidity of the lipid bilayer, and hence the stability, is increased as a result of increasing the content of cholesterol within the of liposomal lipid bilayer [37]. The formation of smaller liposomes with decreasing the cholesterol content could be elucidated based on the enhanced distribution of the aqueous phase within the vesicular liposomes due to the close packing of the lipid bilayer and the improved membrane fluidity. According to the fact that reduced particle size of drug-loading nanocarriers would enhance the cellular uptake of the entrapped cargo, F4 (126.5 ± 3.4) has been chosen for evaluating the effect of liposomal encapsulation on the enhancement of cytotoxic potential of entrapped propolis extract. Release from the selected liposomes exhibits higuchi diffusion kinetics, which is a phenomenological mathematical model that suits the fact that the extract diffuses from the bilayer of such dispersible system. The controlled release strategy of propolis extract proposed by that type of release kinetics would be optimized, in future work, for more therapeutic targets.

In the present study, the effect of encapsulation of propolis within optimized liposomes on Hep-2 cells was investigated and compared to the effect of unencapsulated propolis. The scope of the work was to explore the potential of encapsulation of propolis-loaded liposomes on the enhancement of the antitumor activity of the propolis. The effect of propolis and P-Lip on Hep-2 cell viability was evaluated by MTT assay using serial dilutions of the extract either encapsulated or free.

According to MTT assay results, encapsulated propolis decreased the viability of Hep-2 cells at a much lower concentration than that of propolis extract (IC_50_ of P-Lip is much lower than that of propolis extract), indicating that Hep-2 cells were more sensitive to P-Lip. These findings can be attributed to enhanced cellular uptake and receptor-mediated endocytosis, promoted by the small particle size of formulated liposomes, and then the gradual collapse of the liposomes in the lysosomal apparatus and a posterior intracellular passage of the drug released [38,39]. The smaller particle size of formulated nanocarriers promotes the tendency for the enhanced permeation retention effect (EPR) in the case of IV injection of the formulation with consequent promising targeted in vivo cytotoxic effect [40,41]. However, an MTT assay cannot differentiate whether loss of cell viability is due to apoptosis or necrosis, critical information required to evaluate the efficacy of the antitumor drug

The exact type of cell death may be inferred from morphological changes of the treated cells. Microscopic examination revealed that cells treated P-Lip showed more apoptotic features than those treated with propolis extract. To avoid human errors in detecting apoptotic cells and differentiating them from necrotic cells, a software program was employed to automatically analyze a parameter that is used as an indicator of apoptosis, nuclear area factor.

Morphometric analysis of treated cells revealed that P-Lip produced a much greater reduction in cell circularity and nuclear surface area and, consequently, in nuclear area factor compared with propolis extract. The effect of encapsulated propolis was dose dependent.

Further investigations were performed using flow cytometry assay in order to completely elucidate the molecular mechanisms that participated in the observed cytotoxic activity of propolis and P-Lip. Annexin V not only differentiates between apoptotic and necrotic cells, but it can also determine whether the apoptotic cell in the early or late apoptotic stage by binding to the phosphatidylserine on the surface of apoptotic cells [42]. Flow cytometric results revealed that encapsulated propolis induced apoptosis of Hep-2 cells, and the effect was stronger than that of propolis extract.

Statistical analysis of NAF results showed that both propolis and P-Lip have a statistically significant effect on Hep-2 cells compared with control cells. The effect of propolis-encapsulated liposomes was statistically more significant compared with that of propolis alone.

Prospective studies will be expected to explore and elucidate the molecular mechanisms by which propolis-encapsulated liposomes act on cellular signaling pathways to induce apoptosis.

## 5. Conclusions

In summary, we demonstrated the encapsulation of propolis into liposomes for enhancing its cytotoxic effect against the squamous cell carcinoma cell line (Hep-2) of head and neck. The prepared propolis-encapsulated liposomes have a more cytotoxic effect than unencapsulated propolis. Moreover, the nanosized formulation enhances the delivery and cellular uptake of the apoptotic components of the extract. Morphometric evaluation of the examined cell line (Hep-2) showed that the prepared propolis-encapsulated liposomes produced a much more reduction in the cell circularity and nuclear surface area and consequently in nuclear area factor compared with unencapsulated propolis extract. The cytotoxic effect of propolis-encapsulated liposomes was dose dependent. Liposome is a powerful tool for delivering and enhancing the cytotoxicity of propolis.

## Figures and Tables

**Table 1:** Components and physical properties of the formulated propolis loaded liposomes.

Formula Number	MCL (mmol)	CH%	DL (mg)	EE%	Particle Size (nm)	PDI	Zeta Potential
1	60	40	2.5	72.9 ± 2.8	562.6 ± 13.6	0.521 ± 0.024	−18.3 ± 1.2
2	40	40	7.5	63.2 ± 1.5	185.8 ± 0.4	0.237 ± 0.005	−15.2 ± 2.3
3	80	40	7.5	85.3 ± 3.4	723 ± 20.5	0.654 ± 0.03	−20.2 ± 3.2
4	60	20	2.5	66.5 ± 1.6	126.5 ± 3.4	0.101 ± 0.01	−13.1 ± 1.4
5	60	20	7.5	65.1 ± 2.5	195.3 ± 2.5	0.2 ± 0.05	−16.3 ± 2.5

MCL: Molar concentration of Lipid, CH%: cholesterol percentage to total content, DL: drug loading, PDI: poly dispersity index.

**Table 2:** Stability study of propolis liposomes (F4) after 3 and 6 months of storage at 25 °C.

Formula Number	Zero Time		After 3 Months		After 6 Months	
	EE%	Particle Size (nm)	EE%	Particle Size (nm)	EE%	Particle Size (nm)
F4	66.5 ± 1.6	126.5 ± 3.4	63.3 ± 3.2	142.5 ± 5.6	59.2 ± 5.2	165.3 ± 6.8

**Table 3:** Mathematical modeling of release kinetics.

Formulation	R^2^		
	Zero	First	Higuchi
1	0.931	0.912	0.981
2	0.854	0.846	0.988
3	0.855	0.831	0.975
4	0.813	0.832	0.993
5	0.856	0.964	0.987

**Table 4:** The mean viability percentage of Hep-2 cells treated with decreasing concentrations of propolis for 24 h.

Propolis Concentration (mg/mL)	280	140	35	17.5	8.8	4.4	2.2	1.1	0.275
Viability%	21.43 ± 2.5	26.03 ± 4.5	33.33 ± 3.1	34.13 ± 5.2	38.10 ± 6.1	39.05 ± 5.1	39.37 ± 3.4	72.54 ± 6.1	98.25 ± 5.9

**Table 5:** The mean viability percentage of Hep-2 cells treated with decreasing concentrations of propolis-loaded liposomes and corresponding empty liposomes for 24 h.

Propolis-Containing Liposome Concentration (µg/mL)	1000	500	250	125	64	32	16	8
P-Lip	9.29 ± 3.3	36.41 ± 6.4	65.33 ± 11.3	70.82 ± 7.5	74.78 ± 5.4	79.57 ± 3.5	93.04 ± 2.6	98.97 ± 3.5
Empty Liposomes	92.93 ± 3.6	92.54 ± 5.4	93.47 ± 3.4	92.35 ± 6.1	95.6 ± 4.3	97.31 ± 2.5	96.26 ± 3.4	97.6 ± 2.4

**Table 6:** The mean value of NAF of Hep-2 cells treated with increasing concentrations of propolis (half IC_50_, IC_50,_ and double IC_50_) and liposomal propolis (half IC_50_, IC_50,_ and double IC_50_); the mean value of NAF of control Hep-2 cells was 0.3243.

Propolis	Concentration	Double IC_50_ (3.8 mg/mL)	IC_50_ (1.9 mg/mL)	Half IC_50_ (0.95 mg/mL)
	NAF	0.2283	0.2009	0.1797
Liposomal Propolis	Concentration	Double IC_50_ (0.64 mg/mL)	IC_50_ (0.32 mg/mL)	Half IC_50_ (0.16 mg/mL)
	NAF	0.1509	0.1278	0.1056

**Table 7:** ANOVA test for NAF of propolis treated-Hep-2 (different propolis concentrations) and control cells (24 h after treatment).

	Sum of Squares	Df	Mean Square	F	Sig.
Between Groups	0.122	3	0.041	14.105	<0.0001
Within Groups	0.104	36	0.003		
Total	0.226	39			

**Table 8:** Post hoc comparison test (Bonferroni) for comparison of mean difference of NAF values for different propolis concentrations and control cells (24 h after treatment).

		Mean Difference	Std. Error	Sig.	95% Confidence Interval	
					Lower Bound	Upper Bound
Control	half IC_50_	0.0960324 *	0.0240434	0.002	0.028904	0.163161
	IC_50_	0.1234490 *	0.0240434	<0.000	0.056321	0.190577
	Double IC_50_	0.1445892 *	0.0240434	<0.000	0.077461	0.211718
half IC_50_	IC_50_	0.0274166	0.0240434	1.000	−0.039712	0.094545
	Double IC_50_	0.0485568	0.0240434	0.306	−0.018572	0.115685
IC_50_	Double IC_50_	0.0211402	0.0240434	1.000	−0.045988	0.088269

* Significant when *p* ≤ 0.05 level.

**Table 9:** ANOVA test for the NAF values of different concentrations of P-Lip-treated cells and control cells.

	Sum of Squares	Df	Mean Square	F	Sig.
Between Groups	0.299	3	0.100	153.635	<0.000
Within Groups	0.023	36	0.001		
Total	0.323	39			

**Table 10:** Post hoc comparison test (Bonferroni) of NAF values of different concentrations of P-Lip-treated cells and control cells.

		Mean Difference	Std. Error	Sig.	95% Confidence Interval	
					Lower Bound	Upper Bound
Control	half IC_50_	0.1734342 *	0.0113952	<0.000	0.141619	0.205249
	IC_50_	0.1966242 *	0.0113952	<0.000	0.164809	0.228439
	Double IC_50_	0.2187942 *	0.0113952	<0.000	0.186979	0.250609
half IC_50_	IC_50_	0.0231900	0.0113952	0.296	−0.008625	0.055005
	Double IC_50_	0.0453600 *	0.0113952	0.002	0.013545	0.077175
IC_50_	Double IC_50_	0.0221700	0.0113952	0.357	−0.009645	0.053985

* Significant when *p* ≤ 0.05 level.

**Table 11:** Independent sample *t*-test to compare NAF between propolis and P-Lip at different concentrations.

	Groups	Mean ± SD	*p*-Value
NAF	half IC_50_ Propolis	0.2283 ± 0.0504	
	halfdouble IC_50_ Liposomes	0.1509 ± 0.0092	<0.0001
	IC_50_ Propolis	0.2009 ± 0.0737	
	IC_50_ Liposomes	0.1277 ± 0.0118	0.005
	Double IC_50_ Propolis	0.1797 ± 0.0366	
	Double IC_50_ Liposomes	0.1055 ± 0.0116	<0.0001

## Data Availability

Not applicable.
